# Soluble CD83 Accelerates Wound Healing and Attenuates Inflammatory Responses Induced by Chronic Wound Fluid in a Human 3D in Vitro Wound Healing Model

**DOI:** 10.1111/wrr.70158

**Published:** 2026-04-23

**Authors:** Christian Hollard, Katrin Peckert‐Maier, Moritz Ronicke, Pia Sinner, Fabian Stritt, Tobias Spöttl, Petra Mühl‐Zürbes, Stefan Wirtz, Cornelia Erfurt‐Berge, Alexander Steinkasserer, Dmytro Royzman

**Affiliations:** ^1^ Department of Immune Modulation, Uniklinikum Erlangen Friedrich‐Alexander‐Universität Erlangen‐Nürnberg (FAU) Erlangen Germany; ^2^ Department of Dermatology, Uniklinikum Erlangen FAU Erlangen Germany; ^3^ Department of Medicine 1, Uniklinikum Erlangen FAU Erlangen Germany

**Keywords:** epidermal stem cells, immunomodulation, macrophage polarisation, skin model, tissue regeneration

## Abstract

Chronic wounds, as defined by prolonged inflammation and impaired healing, represent a major and growing healthcare challenge worldwide. Currently, only limited therapeutic options are available to address this condition. This study investigates the potential of soluble CD83 (sCD83), an immunomodulatory molecule, to facilitate wound healing in a human in vitro wound model. This model incorporates keratinocytes, fibroblasts, and macrophages in a three‐dimensional skin construct and was exposed to chronic wound fluid (CWF) to induce an inflammatory and impaired‐healing environment. Treatment with sCD83 resulted in a significant acceleration of wound closure, enhanced macrophage polarisation towards a pro‐regenerative phenotype, and an increase in the production of growth factors such as VEGF and TGF‐β. These effects were also observed under inflammatory conditions induced by CWF, which otherwise impaired healing and sustained a pro‐inflammatory environment. Furthermore, sCD83 enhanced markers associated with epidermal stem cell activity and diminished the expression of pro‐inflammatory mediators, underscoring its immunomodulatory and pro‐regenerative properties within this human 3D in vitro model. Together, these results demonstrate that sCD83 can attenuate inflammatory responses and improve regenerative processes in a CWF‐induced impaired healing environment in vitro. Our findings provide the basis for further preclinical studies to unravel the clinical potential of sCD83 as a novel treatment option for chronic and hard‐to‐heal wounds.

**Abbreviations**
CWF: chronic wound fluid
huDFs: human dermal fibroblasts
huEKs: human epidermal keratinocytes
MΦ: macrophages
PBMCs: peripheral blood mononuclear cells
sCD83: soluble CD83

## 1 Introduction

Chronic wounds are a growing clinical condition, affecting millions of patients worldwide and placing an enormous financial burden on public healthcare systems [1]. These wounds are characterised by delayed wound closure and impaired tissue repair, leading to prolonged hospitalisation, morbidity, and reduced quality of life in patients [2]. Diabetes, pressure ulcers, venous insufficiency, and peripheral arterial disease are common causes of chronic wounds [3]. As the population ages and the incidence of diabetes and obesity increases [4, 5], the number of people with chronic wounds is expected to keep rising sharply.

The development and persistence of chronic wounds involve a complex interplay of cellular and molecular factors, including impaired resolution of inflammation, poor angiogenesis, oxidative stress, and microbial colonisation [6]. Recent literature indicates that immune dysregulation plays an important role in the pathogenesis of chronic wounds, making it an attractive therapeutic target for intervention.

Macrophages (MΦ) play a crucial role in wound healing, since during the inflammatory phase, they help to clear the wound site of dead tissue and pathogens and in addition, secrete pro‐inflammatory cytokines (e.g., IL‐1β, IL‐6 and TNF‐α), to recruit other immune cells [7]. During acute wound healing, pro‐inflammatory MΦ undergo a transition towards a pro‐regenerative phenotype upon the efferocytosis of apoptotic debris. These tissue‐repair MΦ promote the transition towards the proliferative phase by secreting anti‐inflammatory cytokines and growth factors such as IL‐10, TGF‐β and VEGF [8]. Notably, this switch is disturbed in elderly patients and thus associated with wound chronification, a persistent inflammatory milieu and impaired wound healing [9, 10].

A potential therapeutic agent with pro‐regenerative properties in wound healing is the soluble CD83 (sCD83) molecule. sCD83 represents the extracellular domain of CD83, a transmembrane glycoprotein. Originally described as a marker for mature dendritic cells [11, 12], CD83 has subsequently been shown to be also expressed by most activated immune cells, including B and T cells [13] (especially regulatory T cells [14]), neutrophils [15], MΦ [13] and epithelial cells [16, 17, 18]. Notably, when CD83 was depleted in for example, regulatory T cells or MΦ, these cells developed a pro‐inflammatory phenotype as characterised by increased expression of IL‐6, TNF‐α, CXCL1, and G‐CSF [14, 19]. When mice with a conditional knockout for CD83 were challenged within autoimmune models such as experimental autoimmune encephalomyelitis, the mice not only developed an aggravated paralysis but the resolution of inflammation was drastically impaired [20]. Therefore, CD83 acts as an immune checkpoint molecule, which is pivotal for proper cell function and subsequent resolution of inflammation.

In contrast, sCD83 has been shown to promote resolution of inflammation when administered in mouse models of multiple sclerosis [21], rheumatoid arthritis [22] and colitis [23]. It also improved graft survival in corneal transplantation [24, 25], when treated either systemically or after graft incubation. These immunomodulatory effects are mediated by specific tolerogenic mechanisms, including the induction of regulatory T cells and tolerogenic dendritic cells and the polarisation of MΦ towards an alternatively activated, tissue repair phenotype. Furthermore, indoleamine‐2,3‐dioxygenase is crucially involved in the sCD83‐mediated regulatory mechanisms, as blocking experiments using 1‐methyltryptophan completely abolished the protective effects of sCD83 in arthritis and transplantation [22, 24, 25].

Previous work has revealed the pro‐regenerative capacities of sCD83 in a murine full‐thickness wound excision model, resulting in significantly accelerated wound closure, whereby pro‐resolving MΦ played a crucial role [26]. In order to translate these results to the human system, we generated artificial human skin constructs, as established by Gangatirkar et al. [27]. These human skin constructs, consisting of a dermal layer with fibroblasts embedded in collagen and an epidermal layer with keratinocytes, are a well‐established method for specific questions that consider cell‐to‐cell interactions between different cell types in a three‐dimensional manner [27, 28, 29, 30]. By inflicting wounds, these skin constructs are a useful tool for investigating wound healing in an in vitro model [31, 32, 33]. In order to further investigate the role of sCD83‐induced pro‐regenerative MΦ in the human system, we have also implemented human monocyte‐derived MΦ into the human skin constructs as described by Kreimendahl et al. [32]. Wound exudate derived from chronic wounds contains a broad spectrum of inflammatory cytokines, proteinases, bacterial proteins and mediators. These factors have been shown to negatively affect cell viability and regenerative capacity [34, 35, 36]. Therefore, the addition of chronic wound fluid (CWF) to the wound models provides a controlled approach to induce an inflammatory and impaired‐healing environment in vitro.

**FIGURE 1:**
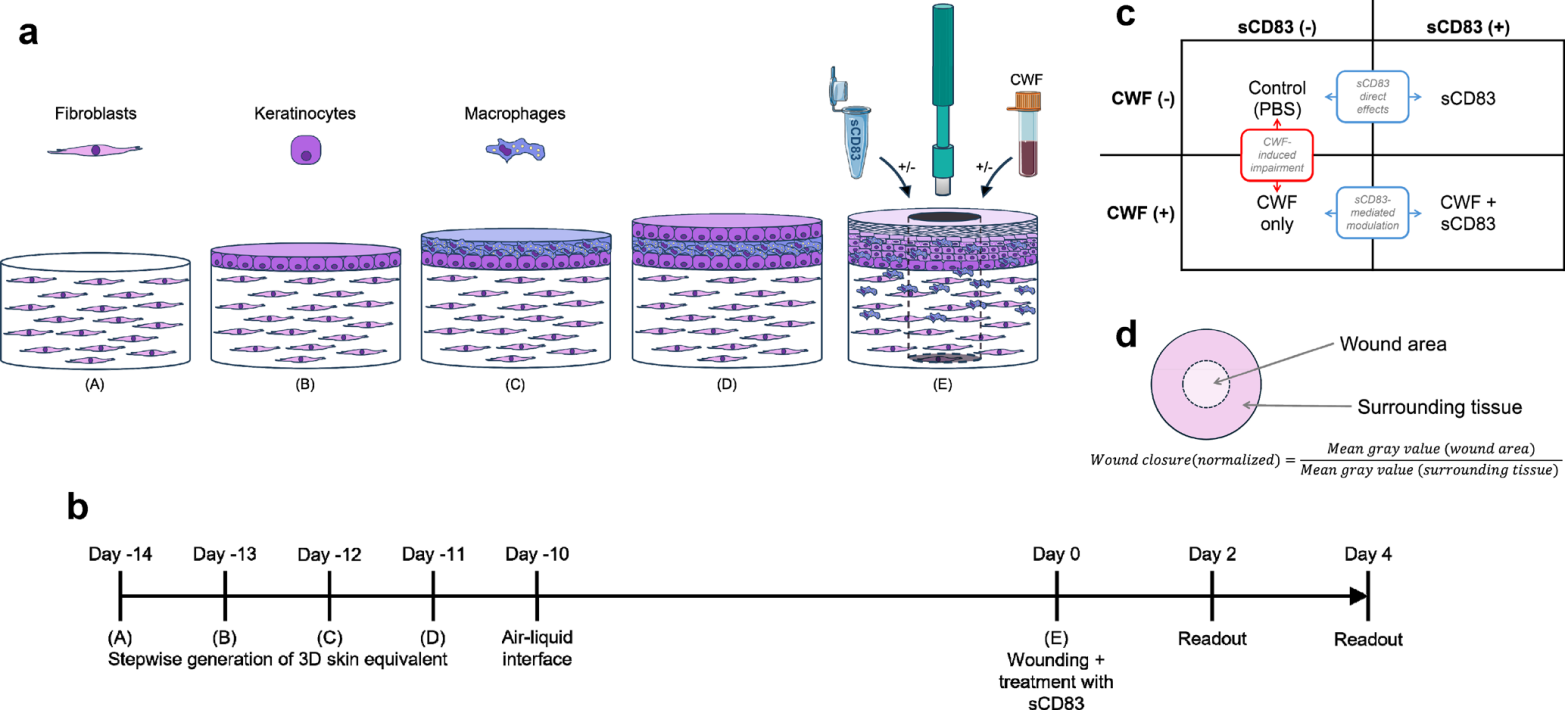
Experimental design of the 3D in vitro wound model. (a) Schematic representation of the 3D skin equivalents. Dermal equivalents– consisting of fibroblasts embedded in a collagen matrix (A). Macrophages sandwiched between two layers of keratinocytes (B, D) (C). To promote cornification, the medium was reduced to form the air‐liquid interface on day −10. After 10 days of keratinocyte differentiation, 3D skin equivalents were wounded—using a 4 mm biopsy punch (E). Treatment (±sCD83; ±chronic wound fluid (CWF)) was initiated immediately after wounding. Samples were collected 2 and 4 days post‐wounding. (b) Experimental timeline. (c) Treatment groups. A 2 × 2 experimental design was applied comparing the effects of CWF exposure and sCD83 treatment: Control (PBS), sCD83 alone, CWF alone and CWF + sCD83. (d) Quantification of wound closure. Wound closure was quantified by normalising the mean grey value of the wound area to that of the surrounding intact tissue.

In this study, we investigated the modulatory potential of sCD83 in a collagen‐based wound healing model containing three different human cell types (i.e., keratinocytes, fibroblasts and MΦ). We analysed changes in wound closure, cell surface markers, cytokine and growth factor levels, and RNA expression profiles. To investigate if and how sCD83 modulates inflammatory responses under impaired‐healing conditions, we supplemented the 3D skin constructs with CWF. Figure 1 illustrates the experimental setup.

## 2 Methods

### 2.1 Cell Isolation and Culture

#### 2.1.1 Human Dermal Fibroblasts and Human Epidermal Keratinocytes

Healthy skin biopsies, obtained from residual tissue from dermatosurgical procedures, were collected, disinfected with alcoholic antiseptic (Schülke) and washed in HBSS (Gibco). To separate the dermis from the epidermis, the skin tissue was incubated overnight at 4°C in 25 U/mL type II dispase (Roche) diluted in HBSS. The reaction was stopped by the addition of an equal volume of 10% FCS (Sigma) in HBSS. The tissue was washed in HBSS before the epidermal layer was peeled off, cut into small pieces with a scalpel, and incubated in 0.5 mg/mL trypsin/0.2 mg/mL EDTA (Sigma) for 10 min at 37°C. After stopping the reaction with 50% FCS in HBSS, the digested tissue was passed through a 100 μm strainer (Falcon) and centrifuged at 1200 rpm for 5 min. The human epidermal keratinocytes (huEKs) were then cultured in KGM‐2 (Promocell) up to passage 4.

Similarly, the dermis was cut into small pieces with a scalpel and digested in 40 U/mL collagenase (Sigma) in HBSS for 90 min at 37°C in an incubator shaker. The reaction was stopped by the addition of an equal volume of FCS. The tissue was passed through a 100 μm strainer and centrifuged at 1200 rpm for 5 min. The human dermal fibroblasts (huDFs) were then cultured in D10 [DMEM (Anprotech) supplemented with 10% FCS, penicillin–streptomycin solution (Sigma), 2 mM pyruvate (Lonza), NEAA (Pan Biotech), and 20 mM HEPES (Anprotech)] up to passage 6.

#### 2.1.2 Human Macrophages

To generate human monocyte‐derived MΦ, peripheral blood mononuclear cells (PBMCs) were obtained from healthy donors using leukocyte reduction system chambers. The PBMCs were then subjected to density gradient centrifugation using Lymphoprep (Nycomed Pharma). The isolated PBMCs were allowed to adhere to 10 cm^2^ dishes (Falcon) for 75 min in adherence medium consisting of RPMI1640 (Lonza), penicillin–streptomycin‐glutamine solution (Sigma), 1% human AB serum (Sigma), and 10 mM HEPES (Lonza). The non‐adherent cell fraction was then removed by multiple rinses of the PBMCs with RPMI1640. Adherent monocytes were cultured in MΦ‐Medium, which included RPMI1640 supplemented with 10% AB serum, penicillin–streptomycin‐glutamine solution, 10 mM HEPES, and 20 ng/mL recombinant human M‐CSF (Peprotech). On day 3, fresh medium and human M‐CSF (20 ng/mL) were added, and on day 6, MΦ were harvested by gentle scraping with a cell scraper (Sarstedt) and used for subsequent experiments.

#### 2.1.3 Generation of Skin Constructs

To build up the dermal equivalent, the huDFs were embedded in a collagen matrix. The cells were trypsinized and counted. For each skin construct, 1 × 10^5^ huDFs were resuspended in 50 μL neutralisation solution and mixed with 450 μL collagen solution (RatCol, Advanced Biomatrix) to obtain a total of 500 μL. The collagen‐cell mixture was then poured into a 12‐well insert (Falcon) using a syringe and cannula and subsequently allowed to jell for 20 min (37°C, 5% CO_2_). The insert was then transferred to a 12‐well plate (Nunc) and covered with 2 mL of D10.

After 24 h in the incubator (37°C, 5% CO_2_), the first layer of huEKs was added. After trypsinization and cell counting, the required amount of huEKs (1 × 10^5^ cells per skin construct) was resuspended in 100 μL medium and applied to the surface of the gels. To allow the cells to adhere, the skin constructs were incubated for 45 min. The skin constructs were then submerged in 2 mL of KGM‐2 medium supplemented with 5% FCS.

After another day, MΦ were added by resuspending 5 × 10^5^ MΦ per skin construct in 100 μL medium and applied to the surface. The skin constructs were then incubated for an additional 45 min to ensure MΦ adherence. Again, the skin constructs were submerged in 2 mL of a mixture of 50% KGM‐2 (adding all supplements) and 50% MΦ‐Medium and cultured for another 24 h at 37°C and 5% CO_2_ before adding a second layer of huEKs as described above.

Finally, after another 24 h of submerged culture, the medium was completely removed and replaced with a mixture of 50% KGM‐2 [supplemented with additional CaCl_2_ (Sigma) to a final concentration of 1.92 mM, and only the supplements insulin, hydrocortisone, epinephrine and transferrin were added] and 50% MΦ‐Medium. An air‐liquid interface was created by adding only 0.8 mL of medium to achieve differentiation of the huEKs. The medium was changed every 2–3 days for an additional 10 days. During this differentiation period, MΦ distributed throughout the skin construct, including the dermal equivalent, as previously demonstrated by immunofluorescence analyses [32]. After this period, the skin constructs were considered fully differentiated and ready for experiments.

### 2.2 Collection of Chronic Wound Fluid

Inclusion criteria for CWF collection were chronic wounds of any aetiology with a duration of more than 12 weeks and the ability of the patient to give consent. There were no changes in the current treatment regimen for the study. Each patient was treated with standard of care according to the current stage of the wound. Samples were collected from a total of 9 patients using sterile swabs (Copan). Swabs (1–4) were used per patient, depending on the amount of exudate. Prior to collection, the wounds were cleaned with a sterile compress moistened with a 0.9% NaCl solution (B. Braun) to remove superficial debris. The swab was then rolled over the entire wound area until a sufficient degree of visual saturation was achieved. The swabs were then immersed in 500 μL of medium. With an uptake capacity of a swab of 150 μL, this results in a concentration of 23% CWF solution. Samples were stored at −80°C, and for subsequent experiments, these samples were thawed, pooled, and sterile filtered (0.45 and 0.22 μm) to obtain a stock solution, aliquots of which were stored at −80°C until use. Table 1 presents the characterisation and demographic data of the patients from whom CWF was collected.

**TABLE 1:** Characteristics and demographics of patients from whom chronic fluid was collected (*n* = 9).

Characteristic	Median (range) *n* (%)
Age (years)	73 (20–89)
Gender	
Female	5/9 (55.6)
Male	4/9 (44.4)
Wound aetiology	
Peripheral arterial disease	2/9 (22.2)
Chronic venous insufficiency	4/9 (44.4)
Mixed ulcer	3/9 (33.3)
Wound characteristics	
Duration (weeks)	78 (26–260)
Area (cm^2^)	71 (21–675)

### 2.3 Wound Model and Treatment

Once the skin constructs were prepared as described above, they were removed from the inserts and wounded using a 4 mm biopsy punch (pfm medical). Skin constructs were placed back into their inserts and the resulting punch holes were filled with acellular collagen. After gelling in an incubator (37°C, 5% CO_2_) for 20 min, 0.8 mL of medium was added and 25% of the medium was replaced on day 2 after wounding. The media were supplemented with sCD83 at a concentration of 25 μg/mL (and 139 μL of the CWF stock solution, resulting in a final concentration of 4% CWF). As the sCD83 protein is solubilised in PBS, the same volume of PBS was added to the control group's media.

Wound models were photographed under a stereo microscope on days 0, 2, and 4. Day 2 was selected to represent the inflammatory phase of wound healing, whereas day 4 was chosen to reflect the proliferative phase within the in vitro model. To determine wound closure, the mean grey value of the wound area was normalised to the mean grey value of the surrounding intact skin construct, as schematically illustrated in Figure 1d. All measurements were performed using ImageJ (version 1.54d).

### 2.4 Flow Cytometry

Samples were collected by removing the skin constructs from the inserts and transferring them to 5 mL 40 U/mL collagenase in HBSS. After 90 min in a shaker at 37°C, the reaction was stopped by adding 5 mL 10% FCS in HBSS. The solution was sieved through a 100 μm strainer and the residue was pressed through the strainer with a syringe stamp. The solution was centrifuged for 3 min (1500 rpm at 4°C) and the cell pellet was resuspended in 50 μL PBS, containing fluorescence‐coupled antibodies purchased from BioLegend, directed against CD14 (FITC; #982502; clone M5E2), CD90 (APC; #328113; clone 5E10), CD206 (PE; #321105; clone 15–2), CD204 (PECy7; #371908; clone 7C9C20), CD11b (BV421; #101236; clone M1/70), CD163 (BV510; #333628; clone GHI/61) and MHCII (APCCy7; #3076018; clone L243). After incubation for 20 min at 4°C, cells were washed with 1 mL PBS (1500 rpm, 3 min, 4°C), resuspended in 150 μL PBS, supplemented with 7AAD (1:1000, Sigma) for live/dead ratio, and analyzed using the FACS Canto II flow cytometer (BD).

### 2.5 Cytometric Bead Array (CBA)

Supernatants of wound models were collected on day 0, day 2, and day 4 after wounding and analysed using the LEGENDplex Human Macrophage/Microglia Panel, LEGENDplex Human Growth Factor Panel (BioLegend), and LEGENDplex Human Inflammation Panel 1, respectively, according to the manufacturer's instructions.

### 2.6 TGF‐β1‐Enzyme Linked Immunosorbent Assay (ELISA)

To determine the TGF‐β1 levels released from the skin constructs into the cell culture supernatants on days 0, 2, and 4 after wounding, a TGF‐β1 ELISA (Invitrogen, #88–8350) was performed according to the manufacturer's instructions.

### 2.7 Histology

On day 4 post‐wounding, skin constructs were collected and preserved in 4% formaldehyde for a minimum of 24 h. The tissue was then processed using conventional histochemical techniques, embedded in paraffin wax, and sectioned at a thickness of 5 μm. The sections were mounted on glass slides, de‐paraffinised, and stained with haematoxylin and eosin (HE), as described before [26].

### 2.8 RNA Extraction, cDNA Synthesis and qPCR Analysis

Cells were extracted as described above (see *Flow cytometry*). Afterwards, cells were lysed by resuspending the pellet in 1 mL RLT buffer (Qiagen), supplemented with 1% β‐mercaptoethanol. RNA was extracted using the RNeasy Plus Micro Kit (Qiagen), according to the manufacturer's protocol. cDNA synthesis was performed according to the instructions of the First Strand cDNA Synthesis Kit (Thermo Fisher). Ribosomal protein L13a (RPL13A) was used as a housekeeping gene. Polymerase chain reaction and quantification were performed using the CFX Opus 96 (Bio‐Rad). The nucleotide sequences of the primer pairs are shown in Table 2.

**TABLE 2:** Primers used for RT‐PCR.

Gene	Sequence forward	Sequence reverse
*RPL13A*	AAAAAGCGGATGGTGGTTCCT	CACGTTCTTCTCGGCCTGTT
*CCL2*	AGCATGAAAGTCTCTGCCGC	TGGTGAAGTTATAACAGCAGGTGAC
*CXCL2*	AAGCTTCCTCCTTCCTTCTGGT	GCCACACTCAAGAATGGGCA
*IL1B*	GCAGAAGTACCTGAGCTCGC	CCTGGAAGGAGCACTTCATCTG
*TNF*	TCAGAGGGCCTGTACCTCAT	GGAGGTTGACCTTGGTCTGG
*KRT6a*	AACCGCATGATCCAGAGGC	CGGCCTGCAGGTTGGC
*KRT14*	GCCATTGATGTCGGCTTCCA	TCTCACAGCCACAGTGGACA

### 2.9 Bioinformatics

RNA was isolated from wound models on day 4 and an additional DNase digestion step was performed to remove residual DNA. RNA sequencing was conducted by Novogene in 2 × 151 paired‐end mode using an Illumina Novaseq 6000 platform. For bioinformatic analysis, the raw fastq reads were processed using the nfcore/rnaseq v3.9 pipeline [37]. TrimGalore v0.6.7 was used for quality control and adapter removal. STAR v2.7.10 [38] and Salmon v1.5.2 [39] were used for splice aware alignment to the reference genomes (mouse: GRCm39, human: GRCh38.107) and BAM‐level quantification. Transcript level estimate files were imported into R 4.4 via tximport v1.28.0 [40]. The R/Bioconductor package DESeq2 v1.40.2 [41] was used for differential gene expression analysis. Ggplo2 was used for generation of graphical illustrations.

### 2.10 Statistical Analysis

All data are presented as mean ± SEM. The normality of the distribution was verified by the Shapiro–Wilk test, and the statistical significance of the results was calculated using either an unpaired *t*‐test, Ordinary one‐way ANOVA for multiple comparisons, or a Mann–Whitney U test, as required for nonparametric distribution. All calculations were performed using GraphPad Prism 8 (GraphPad). P‐values of less than 0.05 were considered to be statistically significant, as marked by asterisks (*p < 0.05, **p < 0.01, ***p < 0.001 and ****p < 0.0001).

## 3 Results

### 3.1 sCD83 Enhances Wound Closure in a MΦ‐Dependent Manner in a Human 3D in Vitro Wound Model

To determine whether the pro‐regenerative effects of sCD83 depend on MΦ, wound closure analyses were first performed in 3D skin constructs generated without MΦ. Under these conditions, sCD83 treatment did not result in a significant enhancement of wound closure compared to PBS controls (Figure S1), suggesting that the presence of macrophages is required for regenerative effects.

**FIGURE 2:**
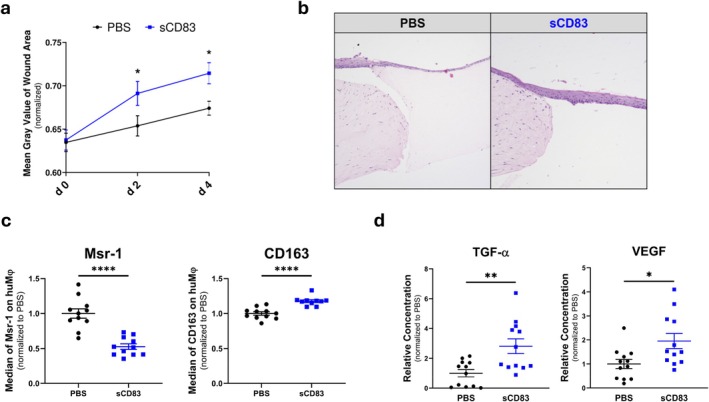
sCD83 accelerates wound healing in a human 3D in vitro wound model. (a) Colorimetric quantification of wound closure using grey values (normalised to mean grey values of 3D skin equivalent). Analyses show a significantly accelerated wound closure in sCD83‐treated samples compared to PBS‐treated controls, on days 2 and 4 (each group and time point *n* ≥ 12). (b) Representative H&E‐stained sections from day 4 reveal a thicker, almost fully restored epidermal layer in sCD83‐treated wounds compared to the incipient re‐epithelialization in PBS controls. (c) Flow cytometric analyses demonstrate a phenotypic switch of macrophages, with decreased expression of the pro‐inflammatory marker Msr‐1 and increased expression of the pro‐regenerative marker CD163, in sCD83‐treated samples (*n* = 11). (d) Analysis of supernatants shows elevated levels of the pro‐healing growth factors TGF‐α and VEGF in sCD83‐treated models (*n* = 12). Statistical analyses: Data are expressed as mean ± SEM. Unpaired *t*‐test. Asterisks mark statistically significant difference (*p < 0.05, **p < 0.01 and ****p < 0.0001).

In Contrast, 3D Wound Models Containing MΦ, Incubated in the Presence of sCD83 After Wounding, Resulted in an Accelerated Wound Closure, When Compared to PBS‐Treated Wounds on Days 2 and 4, as Assessed by the Grey Value Measurement of the Former Wound Site (Figure 2a). Histopathologically, Haematoxylin and Eosin‐Stained Sections Obtained on Day 4 of sCD83 Treatment Showed an Almost Completely Restored Epidermal Layer, Whereas the Wound Area of PBS‐Treated Controls Showed Only Incipient Re‐Epithelialization (Figure 2b). However, no Substantial Fibroblast Infiltration Into the Collagen Plug Was Observed at This Time Point. Since MΦ Play a Crucial Role in the sCD83‐Mediated Effect on Wound Healing [26], the Phenotype of MΦ in the Wound Models Was Analysed by Flow Cytometry. Especially on Day 4, the Presence of sCD83 Enhanced the Transition of MΦ to a Pro‐Regenerative Phenotype, as Indicated by a Significant Downregulation of the Pro‐Inflammatory Surface Marker Msr‐1 and an Upregulation of CD163, a Marker for Pro‐Regenerative MΦ (Figure 2c). This Phenotypic Switch Results in a Pro‐Healing Environment of Growth Factors, as Indicated by Increased Levels of TGF‐Alpha, a Member of the EGF Family, and the Pro‐Angiogenic VEGF (Figure 2d).

#### 3.1.1 Chronic Wound Fluid Induces Pro‐Inflammatory Responses and Impairs Wound Closure in the 3D in Vitro Wound Model

**FIGURE 3:**
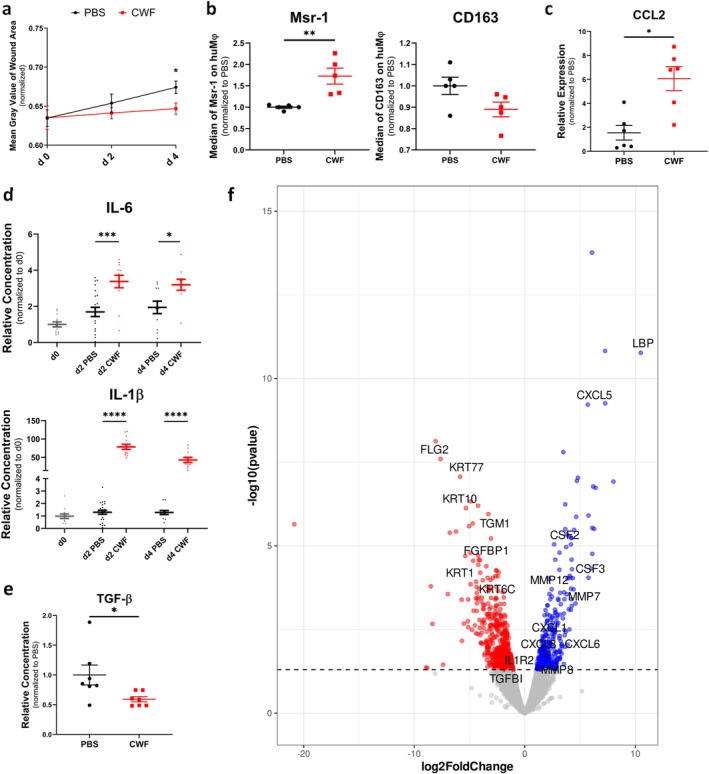
Chronic wound fluid (CWF) induces pro‐inflammatory responses and impairs wound healing in a 3D in vitro wound model. (a) Wound closure analysis on day 4 shows significantly delayed healing in skin constructs treated with 4% CWF compared to controls (each group and time point *n* ≥ 11). (b) Flow cytometry analysis indicates a significantly increased expression of the pro‐inflammatory macrophage marker Msr‐1 and a trend towards reduced CD163 expression in CWF‐treated models (*n* = 5). (c) qPCR analysis demonstrates elevated expression of the pro‐inflammatory chemokine CCL2 in CWF‐treated models (*n* = 6). (d) Cytometric bead array (CBA) reveals higher concentrations of the pro‐inflammatory cytokines IL‐6 and IL‐1β in supernatants of CWF‐treated models (corrected by subtracting the amount added by CWF, as shown in Table 3) (each group and time point *n* ≥ 11). (e) ELISA analysis shows a significant reduction in the concentration of the anti‐inflammatory growth factor TGF‐β in CWF‐treated models (*n* = 7). (f) Volcano plot of RNA sequencing analyses of PBS‐ versus CWF‐treated wound models on day 4 (each *n* = 2). Dots depicted on the right‐hand side of the log2Foldchange value 0 represent significantly upregulated gene transcripts (blue), while the dots on the left‐hand side represent significantly downregulated transcripts (red). Statistical analyses: Data are expressed as mean ± SEM. (a), (e) Unpaired *t*‐test; (b), (c) Mann–Whitney U test; (d) Ordinary one‐way‐ANOVA. Asterisks mark statistically significant difference (*p < 0.05, **p < 0.01, ***p < 0.001, ****p < 0.0001).

To investigate the influence of CWF on wound healing and MΦ phenotypes, the 3D wound models were exposed to media supplemented with 4% CWF. Since the CWF itself contains several cytokines and growth factors (Table 3), the protein concentrations in the supernatants were corrected by subtracting the amount added by CWF. The administration of CWF significantly altered the wound microenvironment, resulting in a pro‐inflammatory phenotype and impaired wound healing (Figure 3a). This inflammatory, non‐healing environment is characterised by induction of pro‐inflammatory MΦ, as indicated by increased expression of Msr‐1 (Figure 3b), elevated levels of IL‐1β and IL‐6 at the protein level (Figure 3d), and CCL2 at the RNA level (Figure 3c). In contrast, levels of the anti‐inflammatory growth factor TGF‐β were reduced (Figure 3f).

**TABLE 3:** Cytokine and growth factor levels in media supplemented with 4% CWF.

Protein	Concentration (pg/mL)
IL‐1β	1461.3
IL‐4	17.3
IL‐6	1080.9
IL‐10	7.9
TNF‐α	58.7
TGF‐α	1.2
VEGF	43.1
TGF‐β	88.4

In addition, RNA sequencing of the CWF‐treated wounds confirmed upregulation of transcripts associated with inflammation such as LBP, CXCL1, CXCL5, CXCL6, CXCL8, CSF2, and CSF3. Matrix metalloproteinases, which are often over‐expressed in chronic wounds [35, 42], were also upregulated. In contrast, skin barrier and epithelialization gene transcripts such as TGM1, FLG2, KRT1, KRT6C, KRT10, and KRT77 were downregulated, resulting in impaired wound healing (Figure 3f).

### 3.2 2sCD83 Restores Pro‐Regenerative Signalling Under CWF‐Induced Inflammatory Conditions

**FIGURE 4:**
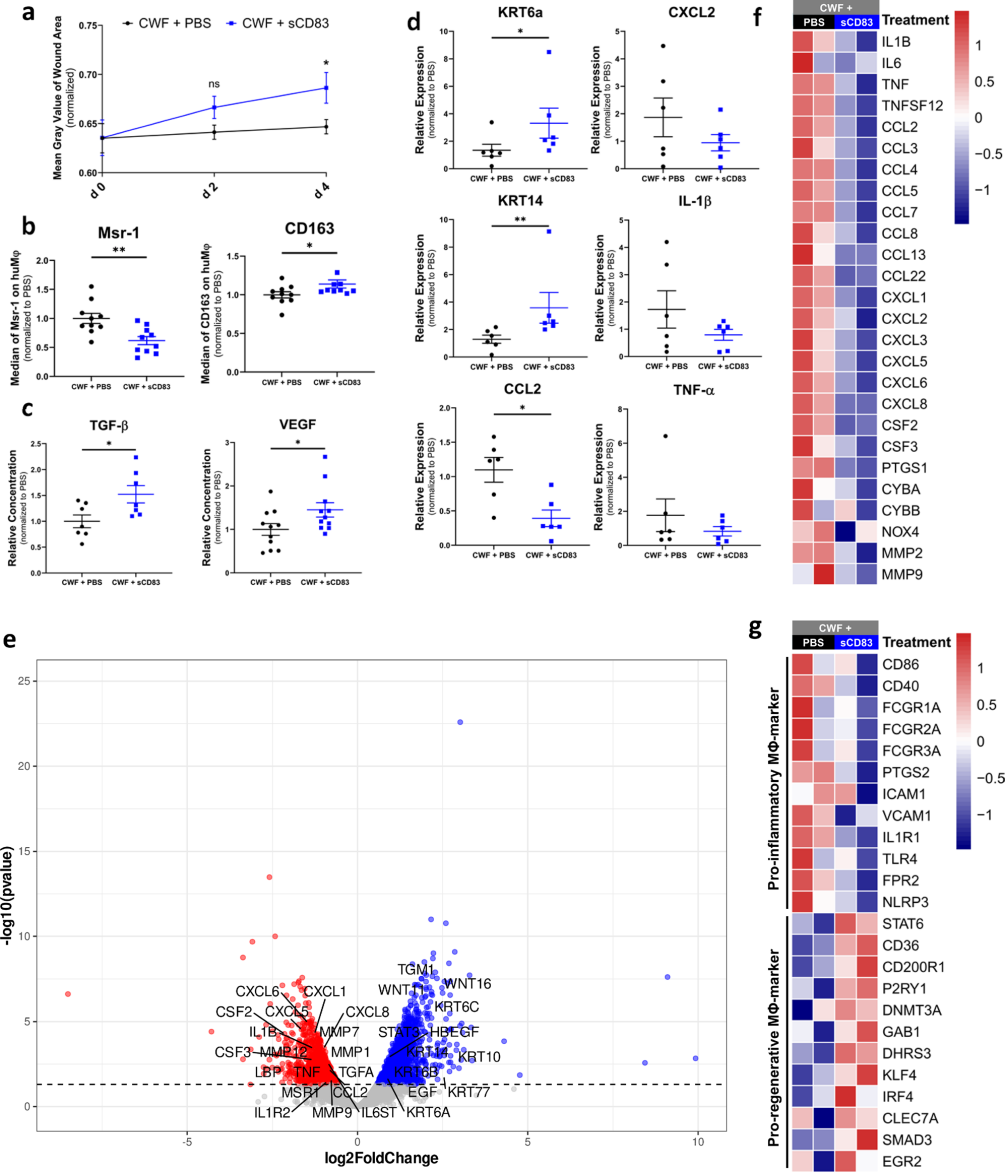
sCD83 attenuates CWF‐induced inflammatory responses and promotes wound healing in a 3D in vitro wound model. (a) Wound closure analyses on day 4, show significantly accelerated healing in CWF‐exposed models, when treated with sCD83 compared to PBS‐controls (each group and time point *n* ≥ 14). (b) Flow cytometry reveals increased expression of the pro‐regenerative macrophage marker CD163 and decreased expression of the pro‐inflammatory marker Msr‐1, in sCD83‐treated models (*n* = 10). (c) Analyses of supernatants demonstrate elevated levels of pro‐healing growth factors such as VEGF (*n* = 11) and TGF‐β (*n* = 7) following sCD83 treatment. (d) qPCR analyses show significant upregulation of cytokeratins KRT6a and KRT14, markers of epithelial stem cell activation and motility, and a reduction in CCL2 expression. Additional pro‐inflammatory gene transcripts (CXCL2, IL1β, TNFα) showed a trend towards reduction (*n* = 6). (e) Volcano plot of RNA sequencing analyses of PBS versus sCD83‐treated CWF‐exposed wound models, on day 4 (each *n* = 2). Dots depicted on the right‐hand side of the log2Foldchange value 0 represent significantly upregulated gene transcripts (blue), while the dots on the left‐hand side represent significantly downregulated transcripts (red). (f), (g) Representative heat maps of differently expressed transcripts in CWF‐exposed wound models of PBS‐ and sCD83‐treated samples on day 4. (f) Represents gene transcripts associated with inflammation and (g) genes for either pro‐inflammatory or pro‐regenerative macrophages. Statistical analyses: Data are expressed as mean ± SEM. (a), (b), (c) Unpaired *t*‐test; (d) Mann–Whitney U test. Asterisks mark statistically significant difference (*p < 0.05, **p < 0.01).

Given that the supplementation of 3D in vitro wound models with CWF resulted in delayed healing and inflammatory response, we were interested to examine whether sCD83 could restore favourable wound healing conditions. It is noteworthy that even under these conditions, sCD83 was able to enhance wound closure (Figure 4a), by promoting a pro‐regenerative MΦ phenotype (Figure 4b). Consequently, the concentration of growth factors, including VEGF and TGF‐β, was elevated in the supernatants of sCD83‐treated skin constructs (Figure 4c). qPCR analysis demonstrated a reduction of the proinflammatory chemokine CCL2 at RNA level (Figure 4d). The activity of epidermal stem cells was induced, as indicated by an increased expression of KRT14 [43]. Additionally, cytokeratins that promote cell motility, such as KRT6a [44], were also induced (Figure 4d). RNA sequencing revealed a downregulation of inflammatory gene transcripts (i.e., IL1B, TNF, CCL2, CXCL1, CXCL5, CXCL6, CXCL8, LBP, CSF2, CSF3) and matrix metalloproteinases (MMP1, MMP7, MMP9, MMP12). On the other hand, sCD83 treatment resulted in an upregulation of WNT11, WNT16 and the epidermal growth factor receptor (EGFR) agonists EGF, TGFA and HBEGF. As a result, cytokeratins (KRT6A, KRT6B, KRT6C, KRT10, KRT14, KRT77) and TGM1—essential for the development of the cornified cell envelope—were upregulated, indicating an accelerated wound closure (Figure 4e). The RNA signature confirmed the anti‐inflammatory properties of sCD83 treatment, as shown in the heatmap with representative pro‐inflammatory gene transcripts (Figure 4f). Furthermore, the sCD83‐induced phenotype is characterised by a transcriptional shift, with reduced expression of pro‐inflammatory and increased expression of pro‐regenerative macrophage signature gene transcripts. (Figure 4g).

## 4 Discussion

Chronic wounds are characterised by impaired healing and persistent inflammation with delayed tissue repair and remodelling. The result is a significant reduction in quality of life and increased morbidity and mortality from complications such as wound infection and amputation. Of note, the five‐year mortality rate of diabetic foot ulcers is comparable to that of common types of cancer [45]. It is also a growing problem for healthcare systems due to the increasing number of patients affected. Chronic wounds are time consuming and costly to treat, resulting in a significant economic impact [1]. Although a wide range of treatment options is available, most current therapies do not effectively address the underlying causes of wound chronicity, such as dysregulated inflammation, cellular senescence, and impaired angiogenesis. While there have been hundreds of new FDA‐approved drugs for the treatment of cancer in recent decades, no new drug for any type of wound healing has reached the clinical stage for more than 25 years [46]. In particular, the use of growth factors seemed promising but disappointed in clinical trials due to the short‐term stimulus and the high level of proteases that degrade growth factors. Thus, unfortunately, they are not the desired game changer [6]. To achieve a more sustained effect, it seems to be more promising to target the cells that are primarily responsible for releasing growth factors, such as MΦ. They secrete a broad variety of growth factors, such as VEGF and TGF‐β, to promote cell proliferation and wound closure [8].

In chronic wounds, the transition from pro‐inflammatory to pro‐regenerative MΦ is disrupted, resulting in persistent inflammation and impaired healing [6, 10]. A promising candidate for promoting this phenotypic switch is sCD83. Among other immunomodulatory effects, it has been shown to induce alternatively activated MΦ, leading to resolution of inflammation and promotion of tissue repair [22, 26, 47]. However, the pro‐regenerative effects of sCD83 have only been demonstrated in a mouse model for acute wounds [26]. Therefore, the aim of this study was to translate these findings into a human in vitro system and to evaluate the pro‐regenerative capacities of sCD83 under the inflammatory conditions induced by CWF. For this purpose, we used primary human‐derived skin cells to generate 3D skin constructs, as already established [27, 28, 29, 30]. Since MΦ play a critical role in wound healing processes and are one of the effector cells of sCD83 treatment, we implemented human monocyte‐derived MΦ into the skin constructs [32]. Previous works have shown that CWF drives pro‐inflammatory cell responses and impairs cell regeneration [34, 35, 36]. CWF represents a complex biological mixture containing inflammatory cytokines, proteases, matrix‐degrading enzymes and bacterial proteins and mediators, all of which may influence cellular behaviour and tissue repair processes. For cell experiments, a concentration of 4% CWF was found to be optimal, demonstrating measurable inflammatory effects without inducing excessive cytotoxicity [48].

In the first part of our experiments, we observed effects consistent with previous findings regarding the pro‐regenerative effects of sCD83 [26]. Wound closure, assessed by measuring the mean grey value in the former wound area, showed a significant cellular accumulation in the acellular matrix, indicating an increased influx of keratinocytes. Given the absence of a significant sCD83 effect in MΦ‐free constructs, we attribute the enhanced wound closure to the induction of pro‐regenerative MΦ as indicated by increased expression of CD163 and decreased expression of Msr‐1. In human MΦ, this implies the induction of alternatively activated pro‐regenerative MΦ [49, 50, 51, 52]. As a result of MΦ‐polarisation, growth factors such as TGF‐α and VEGF were upregulated. TGF‐α is a member of the EGF family and promotes keratinocyte migration during wound healing [53]. VEGF is not only a key player in angiogenesis, but also a promoter of collagen deposition and epithelialization in wounds [54].

Since the transition to pro‐regenerative MΦ is impaired in elderly and diseased patients [55], our findings prompted us to investigate whether sCD83 could modulate regenerative responses under impaired healing responses. To address this, we analysed the effect of CWF on human 3D in vitro skin constructs and evaluated if sCD83 could counteract the CWF‐induced inflammatory alterations. Indeed, CWF impaired the wound closure significantly on day 4 and favoured a pro‐inflammatory MΦ phenotype. This led to a massive inflammatory response with increased levels of CCL‐2, IL‐6 and IL‐1β and decreased secretion of growth factors, such as TGF‐β. Taken together, these findings indicate that exposure to CWF induces an inflammatory and impaired healing environment in the 3D in vitro model, resembling key inflammatory features described in non‐healing wounds [6, 42, 56].

Based on these findings, we aimed to determine whether sCD83 could counteract the pro‐inflammatory and impaired‐healing environment induced by CWF. Remarkably, even under these challenging conditions, sCD83 was able to restore wound closure and mitigate the detrimental effects of CWF. Treatment with sCD83 shifted MΦ to a pro‐regenerative phenotype, as evidenced by increased expression of CD163 and decreased levels of Msr‐1. RNA sequencing confirmed this phenotypic switch and revealed a pro‐regenerative MΦ signature. This phenotypic switch is consistent with its known immunomodulatory mode of action—promoting resolution of inflammation and creating a physiological environment for tissue repair [26, 47]. As a result, EGFR‐targeting growth factors, including EGF, TGF‐α and HBEGF, were upregulated, and activation of the EGFR pathway leads to faster re‐epithelialization [57]. In addition, sCD83 increased levels of critical growth factors such as TGF‐β and VEGF, which are essential for tissue regeneration. TGF‐β plays a key role in modulating immune responses [58] and promoting fibroblast activity, collagen synthesis and epithelialization [56]. VEGF, on the other hand, is essential for angiogenesis, which is necessary for oxygen and nutrient supply during tissue repair [54]. Moreover, RNA sequencing revealed an upregulation of Wnt11 and Wnt16 following sCD83 treatment, two signalling molecules known to be involved in tissue regeneration and wound healing [59, 60]. Thus, we hypothesize that sCD83 exerts its pro‐regenerative effects also by modulating Wnt signalling pathways, further enhancing repair processes by promoting cell proliferation, migration and differentiation. The restoration of these pro‐proliferative pathways in the CWF‐treated in vitro model indicates that sCD83 may mitigate inflammatory barriers that limit regenerative responses under impaired healing conditions. In addition to its effects on MΦ and growth factors, sCD83 also enhanced the activity of epidermal stem cells as indicated by the upregulation of KRT14 and KRT6a. KRT14, a marker of basal keratinocytes, is associated with the regenerative capacity of epidermal stem cells [43], while KRT6a is associated with keratinocyte migration [44], which is crucial for re‐epithelialization. Interestingly, RNA sequencing and qPCR analyses revealed a reduction of pro‐inflammatory mediators such as CCL2, IL‐1β, CXCL2 and TNF‐α in sCD83‐treated models. This supports the notion that sCD83 exerts a broad anti‐inflammatory effect, further facilitating the transition from a CWF‐induced inflammatory state towards a more regenerative profile.

By targeting the underlying immune dysregulation and promoting tissue regeneration, sCD83 offers a novel and versatile approach to address unmet needs in chronic wound care. Our study provides evidence of the therapeutic potential of sCD83 in a human in vitro impaired healing wound model. However, there are some limitations that should be noted. The 3D wound construct, while representative of human skin architecture, lacks the systemic components of wound healing such as vascularization and the broader interactions with the immune system. In addition, histological analyses indicate that wound closure within this model primarily reflects epithelial coverage of the collagen‐filled defect, whereas extensive dermal remodelling and fibroblast‐driven matrix repopulation were not evident within the analysed timeframe. This may relate to the use of primary dermal fibroblasts derived from adult and elderly donors, which are known to exhibit reduced migratory and proliferative capacity compared to younger fibroblast populations [61]. Thus, the model captures early regenerative and immunomodulatory processes but does not fully recapitulate the complex, multi‐layered tissue restoration observed in vivo. Accordingly, in vivo studies will be essential to validate these findings in a more complex patho‐physiological context. In addition, the heterogeneity of chronic wounds, driven by factors such as patient age, comorbidities and wound aetiology, requires further research to evaluate the efficacy of sCD83 in diverse patient populations.

**FIGURE 5:**
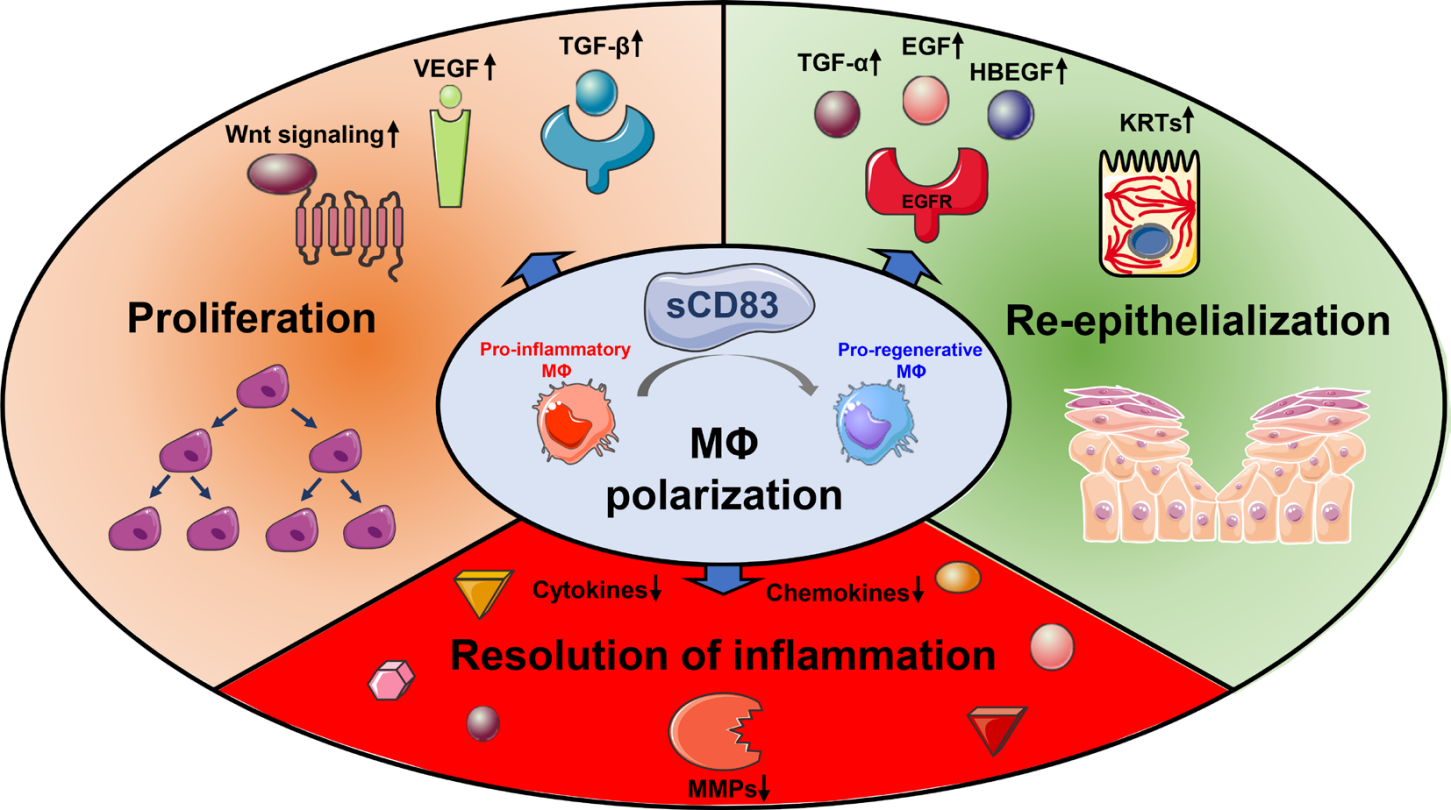
Graphical summary of the proposed mechanisms of sCD83 action in a 3D in vitro impaired‐healing wound model. sCD83 accelerates wound healing under CWF‐induced inflammatory conditions. It promotes the transition of macrophages from a pro‐inflammatory to a pro‐regenerative phenotype, which is a key feature of impaired healing. This phenotypic switch promotes the resolution of inflammation, as indicated by reduced levels of pro‐inflammatory chemokines and cytokines. In addition, the reduction of MMPs prevents excessive matrix degradation. Furthermore, the pro‐regenerative macrophages release signal transduction molecules and activate proliferative pathways, including Wnt, VEGF, and TGF‐β signalling. Increased levels of EGFR ligands promote keratinocyte proliferation and differentiation, as indicated by an increased expression of cytokeratins, leading to a faster re‐epithelialization.

In summary, this study demonstrates that sCD83 modulates inflammatory and regenerative responses in a human 3D in vitro wound model under CWF‐induced impaired healing conditions. A graphical summary of the proposed mechanisms is shown in Figure 5. By inducing MΦ polarisation, it promotes the resolution of inflammation and restores growth factor production, thereby supporting keratinocyte proliferation and activation of epidermal stem cell‐associated markers. sCD83 addresses key barriers for wound healing in an impaired healing and inflammatory environment. These findings provide a strong basis for further research and clinical translation of sCD83 as a potential treatment option for chronic wounds.

## Author Contributions

Conceptualization: Christian Hollard, Dmytro Royzman, Alexander Steinkasserer. Data curation: Stefan Wirtz, Dmytro Royzman. Formal analysis: Christian Hollard, Katrin Peckert‐Maier, Dmytro Royzman. Funding acquisition: Alexander Steinkasserer, Christian Hollard, Moritz Ronicke. Intramural fundings of the Department of Immune Modulation. Investigation: Christian Hollard, Katrin Peckert‐Maier, Moritz Ronicke, Pia Sinner, Fabian Stritt, Tobias Spöttl, Petra Mühl‐Zürbes. Methodology: Christian Hollard, Katrin Peckert‐Maier, Dmytro Royzman. Project Administration: Dmytro Royzman, Katrin Peckert‐Maier. Resources: Cornelia Erfurt‐Berge. Supervision: Alexander Steinkasserer, Dmytro Royzman, Cornelia Erfurt‐Berge. Validation: Stefan Wirtz, Christian Hollard, Katrin Peckert‐Maier. Visualisation: Christian Hollard, Katrin Peckert‐Maier, Dmytro Royzman. Writing – original draft preparation: Christian Hollard. Writing – review and editing: Christian Hollard, Dmytro Royzman, Alexander Steinkasserer, Cornelia Erfurt‐Berge.

## Funding

This work was supported by Initiative Chronische Wunden e.V, Interdisziplinäres Zentrum für Klinische Forschung, Uniklinikum Erlangen, A100, Deutsche Forschungsgemeinschaft, RA 2506/71, TP12, RTG2599, P3, TRR241 A03, WI 3304/8–1.

## Ethics Statement

All experiments using human‐derived tissue have been approved by the institutional ethics commission (No. 22–163‐B) and all participants provided their written informed consent.

## Conflicts of Interest

The authors declare no conflicts of interest.

## Supporting information


**Figure S1:** sCD83 does not enhance wound closure in the absence of macrophages. To assess whether the pro‐regenerative effects of sCD83 are macrophage‐dependent, 3D skin constructs were generated without macrophages. Constructs were wounded and treated with either PBS or 25 μg/mL sCD83 under identical culture conditions. No significant (ns) differences in normalised wound closure were observed between sCD83‐treated and PBS‐treated constructs at the indicated time points (*n* = 4 per group). Data are presented as mean ± SEM. Statistical analysis was performed using an unpaired *t*‐test.

## Data Availability

Raw sequencing data will be available from the European Nucleotide Archive (ENA) via the accession number PRJEB95895. The authors affirm that all other data necessary for confirming the conclusions of the article are present within the article, figures, and tables.
