# Supplementary material for: Soluble CD83 Accelerates Wound Healing and Attenuates Inflammatory Responses Induced by Chronic Wound Fluid in a Human 3D in Vitro Wound Healing Model
Source: Wound Repair Regen. 2026 Apr 23;34:e70158. doi: 10.1111/wrr.70158 (PMC13106732; doi:10.1111/wrr.70158)
Supplement: Supplementary file 1 — Figure S1: sCD83 does not enhance wound closure in the absence of macrophages. To assess whether the pro‐regenerative effects of sCD83 are macrophage‐dependent, 3D skin constructs were generated without macrophages. Constructs were wounded and treated with either PBS or 25 μg/mL sCD83 under identical culture conditions. No significant (ns) differences in normalised wound closure were observed between sCD83‐treated and PBS‐treated constructs at the indicated time points (n = 4 per group). Data are presented as mean ± SEM. Statistical analysis was performed using an unpaired t‐test. [file WRR-34-0-s001.pptx]

## Slide 1
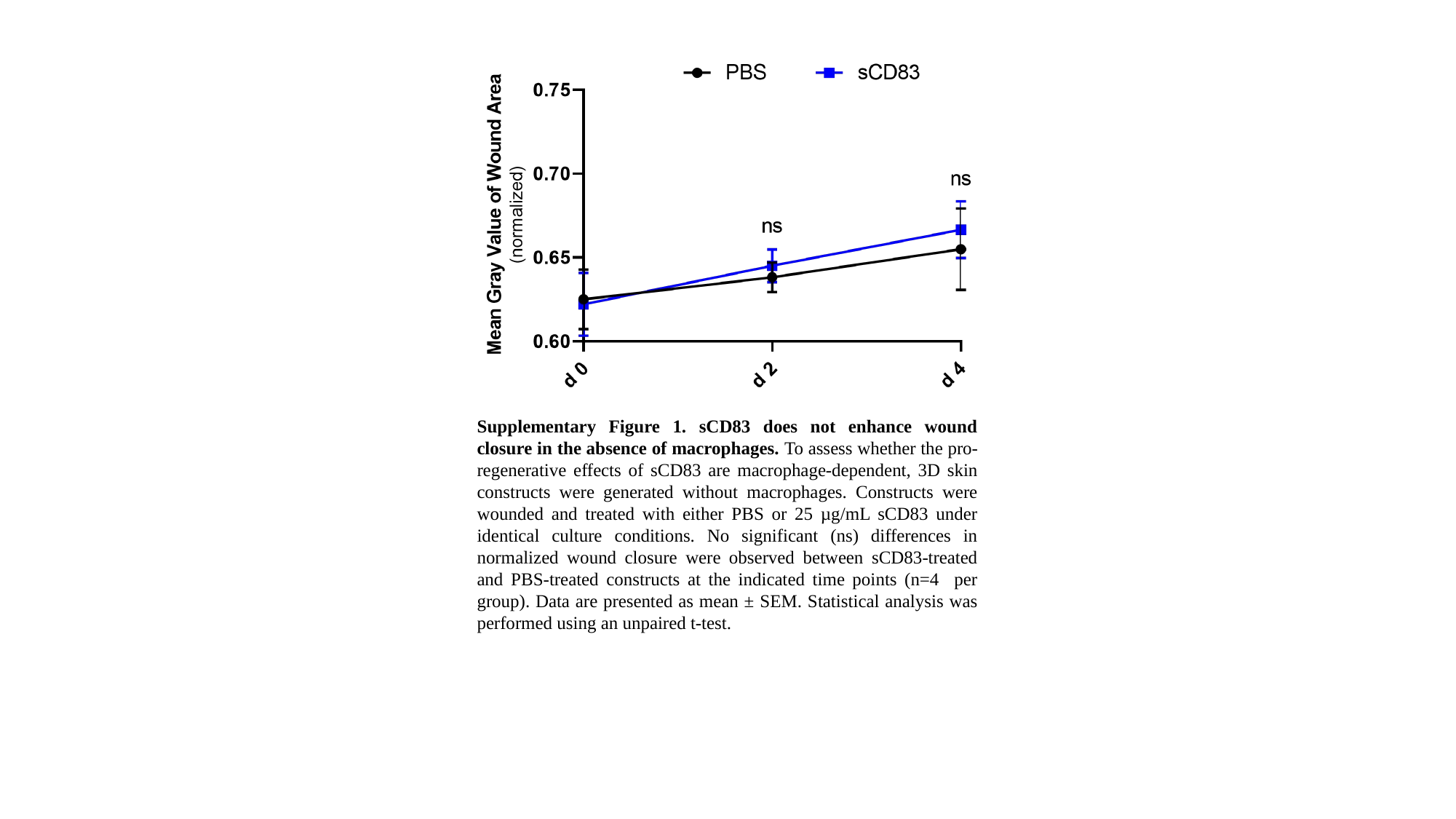

Supplementary Figure 1. sCD83 does not enhance wound closure in the absence of macrophages. To assess whether the pro-regenerative effects of sCD83 are macrophage-dependent, 3D skin constructs were generated without macrophages. Constructs were wounded and treated with either PBS or 25 µg/mL sCD83 under identical culture conditions. No significant (ns) differences in normalized wound closure were observed between sCD83-treated and PBS-treated constructs at the indicated time points (n=4 per group). Data are presented as mean ± SEM. Statistical analysis was performed using an unpaired t-test.
